# Broad‐coil Repetitive Transcranial Magnetic Stimulation (rTMS) of the Precuneus in Alzheimer’s Disease

**DOI:** 10.1002/alz70859_098913

**Published:** 2025-12-25

**Authors:** Michael K Leuchter, Hanadi Oughli, Kelly A. Durbin, Nicholas Jackson, David Elashoff, Timothy S Chang, Juliana Corlier, Julia Sun, Doan Ngo, Cole Matthews, Darice Y. Wong, Brent L. Fogel, Gal Bitan, Andrew F Leuchter, Keith Vossel, Nanthia Suthana

**Affiliations:** ^1^ University of California, Los Angeles, Los Angeles, CA USA; ^2^ UCLA, Los Angeles, CA USA; ^3^ Movement Disorders Programs, Department of Neurology, David Geffen School of Medicine, University of California, Los Angeles, Los Angeles, CA USA

## Abstract

**Background:**

Brain network dysfunction, particularly within the default mode network (DMN), is an increasingly apparent manifestation of Alzheimer’s Disease (AD) progression. Repetitive transcranial magnetic stimulation (rTMS) can target key DMN hubs, maintain signaling function, and delay or improve clinical outcomes in AD. We present here the rationale, design, and preliminary open‐label data of a study using off‐the‐shelf equipment and the latest clinical evidence to expand on prior rTMS work and reduce participant burden in the process.

**Methods:**

We are conducting a two‐stage trial of large‐coil rTMS targeting the precuneus (a key hub in the DMN affected by AD) in 54 participants with mild to moderate Alzheimer’s Clinical Syndrome focused primarily on determining tolerability and feasibility and secondarily studying short‐term efficacy for episodic memory. The currently active first stage involves 5‐10 participants receiving open‐label treatment for protocol refinement. The subsequent stage will consist of a 1:1 randomized, double‐blind, sham controlled clinical trial to study feasibility and tolerability while exploring target engagement and short‐term efficacy for memory. Participants will undergo 16 total rTMS brain stimulation sessions over the course of 5 weeks. A full open‐label extension treatment course will be offered to the sham group after unblinding. Outcomes focus on completion rates and adverse events to demonstrate feasibility and tolerability. Further exploratory outcomes will include neuropsychological assessments, electroencephalography (EEG), neuroimaging, and blood biomarkers to demonstrate feasibility of collection and explore preliminary changes in these measures.

**Results:**

We anticipate this treatment is feasible and tolerable and may show evidence of target engagement and clinical improvement. We plan to present current preliminary open‐label data as analyzed at AAIC.

**Conclusion:**

Should we achieve expected positive outcomes in feasibility and tolerability, this will justify future work focusing on clear demonstrations of clinical efficacy and biomarker engagement, as well as enhancement of generalizability and scalability.

Trial Registration: Clinicaltrials.gov NCT06597942

